# Automated Classification of Lymphoma Subtypes From Histopathological Images Using a U-Net Deep Learning Model: Comparative Evaluation Study

**DOI:** 10.2196/72679

**Published:** 2026-01-06

**Authors:** Jin Zhao, Xiaolian Wen, Li Ma, Liping Su

**Affiliations:** 1 Department of Hematology, Cancer Hospital Affiliated to Shanxi Medical University, Shanxi Province Cancer Hospital, Shanxi Hospital Affiliated to Cancer Hospital, Chinese Academy of Medical Sciences, No. 3, Zhigong New Street, Taiyuan, 030013, China, 86 0351-4650984

**Keywords:** deep learning, lymphoma, U-Net, pathological subtype, automated diagnosis, medical image analysis, artificial intelligence, AI

## Abstract

**Background:**

Accurate classification and grading of lymphoma subtypes are essential for treatment planning. Traditional diagnostic methods face challenges of subjectivity and inefficiency, highlighting the need for automated solutions based on deep learning techniques.

**Objective:**

This study aimed to investigate the application of deep learning technology, specifically the U-Net model, in classifying and grading lymphoma subtypes to enhance diagnostic precision and efficiency.

**Methods:**

In this study, the U-Net model was used as the primary tool for image segmentation integrated with attention mechanisms and residual networks for feature extraction and classification. A total of 620 high-quality histopathological images representing 3 major lymphoma subtypes were collected from The Cancer Genome Atlas and the Cancer Imaging Archive. All images underwent standardized preprocessing, including Gaussian filtering for noise reduction, histogram equalization, and normalization. Data augmentation techniques such as rotation, flipping, and scaling were applied to improve the model’s generalization capability. The dataset was divided into training (70%), validation (15%), and test (15%) subsets. Five-fold cross-validation was used to assess model robustness. Performance was benchmarked against mainstream convolutional neural network architectures, including fully convolutional network, SegNet, and DeepLabv3+.

**Results:**

The U-Net model achieved high segmentation accuracy, effectively delineating lesion regions and improving the quality of input for classification and grading. The incorporation of attention mechanisms further improved the model’s ability to extract key features, whereas the residual structure of the residual network enhanced classification accuracy for complex images. In the test set (N=1250), the proposed fusion model achieved an accuracy of 92% (1150/1250), a sensitivity of 91.04% (1138/1250), a specificity of 89.04% (1113/1250), and an *F*_1_-score of 90% (1125/1250) for the classification of the 3 lymphoma subtypes, with an area under the receiver operating characteristic curve of 0.95 (95% CI 0.93‐0.97). The high sensitivity and specificity of the model indicate strong clinical applicability, particularly as an assistive diagnostic tool.

**Conclusions:**

Deep learning techniques based on the U-Net architecture offer considerable advantages in the automated classification and grading of lymphoma subtypes. The proposed model significantly improved diagnostic accuracy and accelerated pathological evaluation, providing efficient and precise support for clinical decision-making. Future work may focus on enhancing model robustness through integration with advanced algorithms and validating performance across multicenter clinical datasets. The model also holds promise for deployment in digital pathology platforms and artificial intelligence–assisted diagnostic workflows, improving screening efficiency and promoting consistency in pathological classification.

## Introduction

Lymphoma is a malignant tumor originating in the lymphatic system, with a steadily increasing global incidence. On the basis of its clinical features and histological characteristics, lymphoma can be classified into various subtypes, with Hodgkin lymphoma and non-Hodgkin lymphoma being the most prevalent [1,2]. Accurate classification and grading of lymphoma are critical for clinical treatment and prognostic evaluation [3]. Conventional diagnostic approaches rely on the visual examination of tissue sections by pathologists under a microscope, a process that is labor-intensive and influenced by subjective judgment. Diagnostic accuracy is often limited by the pathologist’s expertise and technical proficiency and by sample quality [4,5]. With the advancements in medical imaging and computational technology, artificial intelligence (AI) techniques such as deep learning have been gradually introduced into medical image analysis [6], showing great potential in automated image segmentation, feature extraction, and disease diagnosis [7].

Deep learning, particularly convolutional neural networks (CNNs), has achieved significant success in medical image analysis, offering new approaches for automated lymphoma diagnosis [8,9]. Among them, the U-Net architecture, a specialized form of convolutional neural network, has demonstrated notable effectiveness in segmentation tasks by enabling precise delineation of lesion areas and producing high-quality inputs for subsequent classification and grading [7,10]. Despite these advancements, several challenges persist in the automated analysis of lymphoma histopathology images [11]. Minimal morphological differences between subtypes and the inherent complexity of lesion regions increase classification difficulty [12]. Moreover, the performance of existing deep learning models remains highly dependent on large volumes of annotated data. Limited datasets and overfitting remain a pressing challenge [13,14]. Therefore, developing a deep learning model that can effectively improve the accuracy of lymphoma pathological image classification and grading has become a critical research direction in medical image analysis [15].

Recent studies have shown that pathological diagnosis of lymphoma is associated with considerable interobserver variability, with consistency coefficients (κ values) ranging between 0.55 and 0.70. Misclassification frequently occurs among morphologically similar subtypes, including follicular lymphoma (FL) and mantle cell lymphoma (MCL). Previous research has applied CNNs and other machine learning models for automatic classification of pathological images, such as Inception-V3–based models for breast cancer image analysis or residual network (ResNet)–based models for predicting lung cancer types and molecular features. These studies highlight the substantial potential of AI in assisting pathological diagnosis. However, research specifically focused on the classification of lymphoma pathological subtypes remains limited and often restricted to individual subtypes or single-model architectures. There is still a lack of systematic validation of hybrid deep learning models for fine-grained classification across multiple lymphoma subtypes.

This study aimed to achieve automated segmentation, subtype classification, and grading of lymphoma pathological images by integrating deep learning techniques, including the U-Net model, attention mechanisms, and residual networks (ResNet), all of which are established deep learning approaches [4]. This research has significant clinical and practical implications. Deep learning models have the potential to significantly improve the accuracy of lymphoma pathological diagnosis, reduce human-related diagnostic errors, and provide more objective and consistent diagnostic results [16]. Automated systems can also process large volumes of pathological slides at high speed, offering an efficient auxiliary tool for pathologists, accelerating the lymphoma diagnostic workflow, and reducing patient waiting times. Moreover, precise identification and grading of lymphoma subtypes may contribute to the development of personalized treatment strategies, allowing for more tailored treatment plans and improving treatment outcomes and survival rates [17].

The primary objective of this study was to explore and evaluate the application of deep learning techniques in classifying and grading lymphoma pathological subtypes. An automated image analysis system was constructed based on the U-Net architecture. Through deep learning methods such as image segmentation, feature extraction, and classification prediction, this study sought to accurately identify different lymphoma subtypes and achieve effective grading. In addition, this study used cross-validation techniques to assess the stability and accuracy of the proposed model. A comparative analysis with traditional pathological diagnostic methods was conducted to verify the model’s clinical feasibility. Ultimately, this study aimed to provide effective technical support for the early diagnosis of lymphoma, advancing the application and development of AI into the field of medical pathology.

## Methods

### Data Collection and Preprocessing

A total of 620 high-quality histopathological images representing 3 major lymphoma subtypes were collected from The Cancer Genome Atlas (TCGA) and The Cancer Imaging Archive (TCIA). The number of samples per subtype was approximately balanced. Inclusion criteria required complete pathological annotations and clearly defined tissue structures. Images exhibiting severe artifacts, incomplete labeling, or low resolution were excluded. All slides were stained with hematoxylin and eosin and resized to 512 × 512 pixels to ensure model compatibility. No stain normalization was applied.

The image data in this study primarily consisted of standardized lymphoma tissue section images captured using high-resolution digital scanners and stored in TIFF or PNG format. Image preprocessing steps included denoising, contrast enhancement, and image normalization. Gaussian filtering was used to reduce noise, whereas histogram equalization was used to enhance contrast and improve the visibility of lesion regions. All images were uniformly cropped and resized to ensure consistent input data quality for the network. These preprocessing procedures were implemented to improve data quality and model classification accuracy (Figure S1 in Multimedia Appendix 1).

### Feature Extraction From Image Data

This study used the U-Net architecture to conduct image segmentation, enabling precise identification of lesion areas and the generation of high-quality segmentation maps. Subsequently, ResNet was used for feature extraction and classification. The residual learning structure in ResNet enabled the capture of deep pathological features from the segmented images, thereby improving classification accuracy and supporting automated grading of lymphoma subtypes. During training, 5-fold cross-validation was used to optimize model hyperparameters and reduce the risk of overfitting. The Adam optimization algorithm was used in conjunction with a learning rate decay strategy to enhance training stability and convergence (Figure S2 in Multimedia Appendix 1). The training dataset was randomly divided into 3 subsets, with 70% allocated to the training set and 15% each allocated to the validation and test sets.

### Model Construction and Training

The model used in this study was based on the standard U-Net architecture, enhanced by integrating ResNet residual modules and attention mechanisms. To achieve subtype classification and grading of lymphoma, the combined use of U-Net and ResNet allowed for effective image segmentation and deep feature extraction. The cross-entropy loss function was used, and the Adam optimizer with an initial learning rate of 0.001 was adopted to ensure stable model convergence. A learning rate decay mechanism was applied to progressively adjust the learning rate, improving model convergence and training stability (Figure S3 in Multimedia Appendix 1). The model can automatically extract and learn features of different lymphoma subtypes and grades from images through this training process, enabling efficient classification and accurate grading of new images.

To further justify the selected model architecture, a comparative evaluation was conducted against several classic CNN frameworks widely used in medical image analysis (Table S1 in Multimedia Appendix 1). Fully convolutional networks offered advantages in simplicity and computational efficiency but demonstrated limited sensitivity in detecting small lesions and boundary structures in pathological images. SegNet achieved reliable semantic segmentation performance but often resulted in detail loss during the upsampling stage due to its deconvolutional design. DeepLabv3+, which uses atrous convolution and multiscale feature fusion, significantly improved segmentation accuracy but introduced increased model complexity, computational cost, and reduced interpretability in clinical settings. In contrast, the hybrid deep learning framework proposed in this study—featuring a U-Net backbone, ResNet residual connections, and attention mechanisms—achieved a better balance among segmentation accuracy, training stability, and interpretability. It is particularly well-suited for the classification and grading of lymphoma images characterized by blurry boundaries and high subtype heterogeneity.

### Model Evaluation and Optimization

Model performance was evaluated using multiple metrics, including accuracy, recall, precision, and *F*_1_-score, to assess classification effectiveness. The receiver operating characteristic curve and the area under the curve (AUC) were used to further evaluate the model’s discriminative ability. To prevent overfitting and enhance generalization capability, the model incorporated dropout layers and data augmentation techniques. Augmentation strategies included image rotation, translation, and flipping, which increased the diversity of the training data and improved model robustness. During training, the performance on the validation set was monitored in real time to identify and preserve the model with the best generalization ability for subsequent testing (Figure S4 in Multimedia Appendix 1).

### Traditional Pathology Baseline and Metric Sources

To establish a reproducible baseline for comparison with traditional pathological diagnosis, we conducted a randomized crossover washout reading study. Six board-certified pathologists with ≥5 years of professional experience were enrolled. From the 620 cases included in this study, a subset of 180 (29%) was stratified by subtype, center, and diagnostic difficulty to form the reading set. The reference standard (ground truth) was determined by 2 senior pathologists independently, with any discrepancies resolved by a third expert adjudicator. Each reader completed case interpretation under 2 study arms: unassisted and AI-assisted (with U-Net–generated segmentation masks and posterior probability and attention cues displayed). The order of arms and case sequence was computer randomized, with a washout period of at least 2 weeks, and the readers were blinded to previous assessments. The primary endpoint was case-level accuracy. Secondary endpoints included sensitivity, specificity, *F*_1_-score, AUC, mean average precision (mAP), weighted κ, and single-case reading time. Segmentation performance was evaluated using case-level dice coefficient and intersection over union (macro- and microaveraged). Classification and grading were assessed at the whole-slide image level; patch-level probabilities were aggregated via attention pooling, and classification thresholds were determined on the validation set using the Youden index and subsequently fixed for the test set. For statistical analyses, the DeLong test was used to compare AUCs, whereas the McNemar test was applied to paired proportions (accuracy, sensitivity, and specificity). The 95% CIs were estimated via the bias-corrected and accelerated bootstrap method (1000 iterations), and Holm-Bonferroni correction was used for multiple comparisons.

### Image Data Analysis Methods

Image data analysis focused on 2 primary tasks: image classification and subtype grading. First, deep CNNs based on U-Net and ResNet were used to analyze histopathological slide images. These architectures effectively captured cellular morphology and tissue-level structural features, enabling accurate differentiation among lymphoma subtypes. For subtype grading, the analysis extended beyond subtype identification to include the evaluation of lymphocyte distribution, morphological variation, and lesion heterogeneity within the image (Figure S5 in Multimedia Appendix 1). To ensure accuracy in classification and grading, the image analysis methods integrated traditional medical imaging analysis techniques with deep learning approaches using automated and manual annotation strategies.

### Statistical Analysis and Result Validation

Following model construction, statistical methods were used to validate its performance comprehensively. In addition to conventional classification evaluation metrics, a confusion matrix was used to analyze the model’s prediction results in detail, identifying errors such as false positives and false negatives. The average accuracy of the model was calculated through multiple repeated experiments, and k-fold cross-validation was used to ensure the robustness of the results. Furthermore, 2-tailed *t* tests or ANOVA were conducted to analyze differences among models, ensuring that the selected model demonstrated superior performance in lymphoma subtype classification and grading tasks (Figure S6 in Multimedia Appendix 1).

### Feature Analysis and Subtype Differentiation

Feature analysis was conducted using the U-Net and ResNet architectures to extract and differentiate cellular and tissue-level characteristics from histopathological images. Key features, including tumor cell morphology, tissue architecture, and cellular distribution, were effectively captured to support the accurate identification of lymphoma subtypes and enhance understanding of histological variation among them.

U-Net provided precise segmentation of lesion regions, whereas ResNet enabled efficient feature extraction and supported deeper network training. The combination of these 2 architectures facilitated the recognition of complex spatial relationships between cells and tissues, contributing to improved classification accuracy.

In addition, statistical analysis methods are crucial in evaluating model performance. Metrics such as sensitivity, specificity, precision, and recall were calculated to comprehensively assess the performance of different models in classifying lymphoma pathological subtypes. Confusion matrices and receiver operating characteristic curve analyses were also used to evaluate each model’s predictive effectiveness, providing deeper insights into the strengths and limitations of the models in identifying various subtypes.

### Ethical Considerations

All histopathological images used in this study were obtained from publicly available databases (TCGA project and TCIA). All datasets underwent strict anonymization procedures before public release and complied with the relevant ethical policies and data sharing regulations. No new patient samples were collected during the course of this research. Therefore, additional ethics approval was not required. This study adhered to the ethical principles outlined in the Declaration of Helsinki and its subsequent amendments. Access to the image data is available under the open access policies of TCGA and TCIA or upon request from the corresponding author.

## Results

### Effect Analysis of Data Preprocessing and Image Quality Enhancement

**Figure 1.**
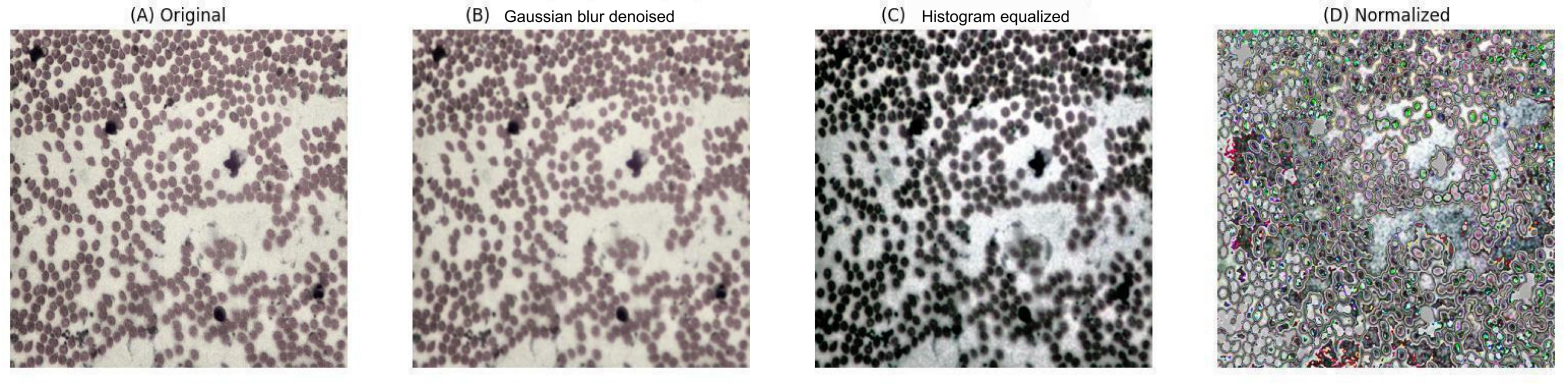
Visualization of data preprocessing effects. This figure showcases the effects of different preprocessing stages on the same lymphoma image slide: (A) the original image; (B) the denoised image processed through Gaussian filtering; (C) the contrast-enhanced image after histogram equalization; and (D) the normalized image, showing clearer edges and details.

In this study, the quality of lymphoma pathological slide images directly affected the performance of the deep learning model. Therefore, multiple image preprocessing techniques, including Gaussian filtering for noise reduction, histogram equalization, and image normalization, were applied to improve image quality. Figure 1 illustrates the visual differences between raw and preprocessed images. In the preprocessed images, background noise was substantially reduced, edges appeared sharper, and lesion regions became more prominent. These enhancements facilitated clearer visualization of pathological features and improved input consistency for model training. During subsequent deep learning model training, the preprocessed images exhibited higher segmentation accuracy and lower error rates. A detailed dataset analysis was also conducted to assess sample distribution. Table 1 presents the number of samples in the training, validation, and test sets, along with the specific distribution of each class, providing crucial data support for subsequent model training.

**Table 1.** Distribution of dataset samples—the number of samples in the training, validation, and test sets along with their corresponding categories.

Dataset	Number of immune cell samples	Number of epithelial cell samples	Number of matrix cell samples	Total
Training set	4000	3000	2500	9500
Validation set	500	400	350	1250
Test set	500	400	350	1250
Total	5000	3800	3200	12,000

To evaluate the impact of image preprocessing on model performance, a comparative experiment was conducted using both unprocessed and preprocessed images. The results showed that unprocessed images achieved only 77.12% (964/1250) accuracy in the segmentation task, with poor edge detection quality, slower model convergence, and a substantially higher error rate. In contrast, preprocessed images achieved a segmentation accuracy of 84.4% (1055/1250), demonstrating clearer delineation of lesion boundaries and exhibiting a noticeably faster training speed (Table S2 in Multimedia Appendix 1). These findings highlight the importance of preprocessing steps such as Gaussian filtering, histogram equalization, and normalization in significantly enhancing segmentation performance.

### Performance of the U-Net Model in Image Segmentation

**Figure 2.**
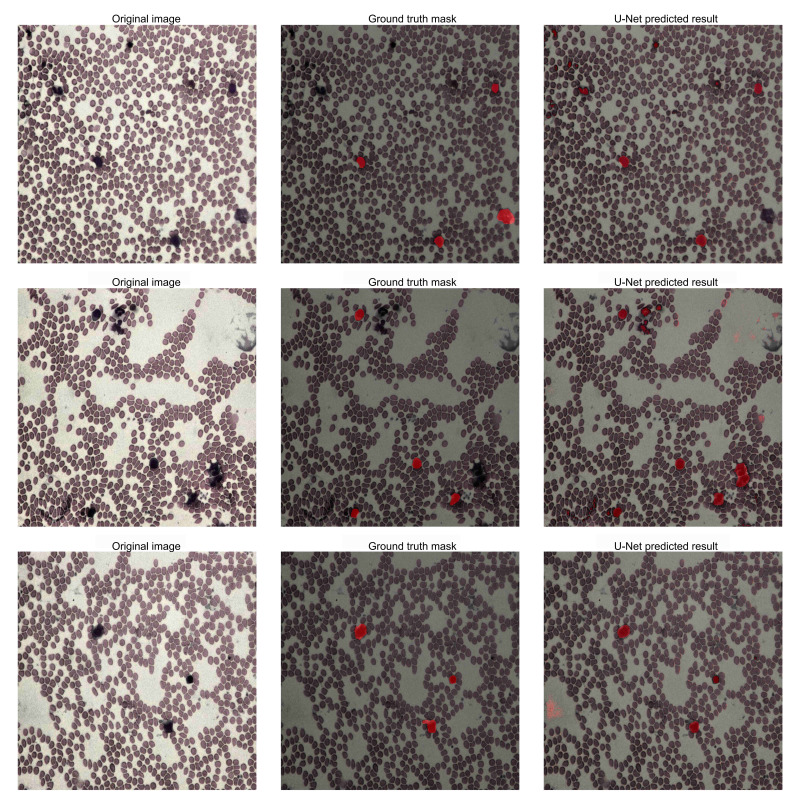
U-Net image segmentation results. This figure shows the performance of the U-Net model on the image segmentation task for lymphoma pathological slides. Segmented regions are marked in different colors to distinguish lesion areas from healthy tissue.

The application of the U-Net architecture in image segmentation demonstrated strong performance in accurately segmenting lesion areas in lymphoma pathological slides, significantly outperforming traditional methods. Figure 2 illustrates the performance of the U-Net model in segmentation tasks. During training and validation, the U-Net model achieved a segmentation accuracy exceeding 85.04% (1063/1250) on the test set, with a recall rate of 88% (1100/1250). These results confirmed the capability of U-Net in handling complex medical images, particularly lymphoma pathological slides. Effective segmentation of lesion areas provided high-resolution input data for downstream tasks, including subtype classification and pathological grading.

### Analysis of the Synergistic Effects of Attention Mechanisms and ResNet

**Figure 3.**
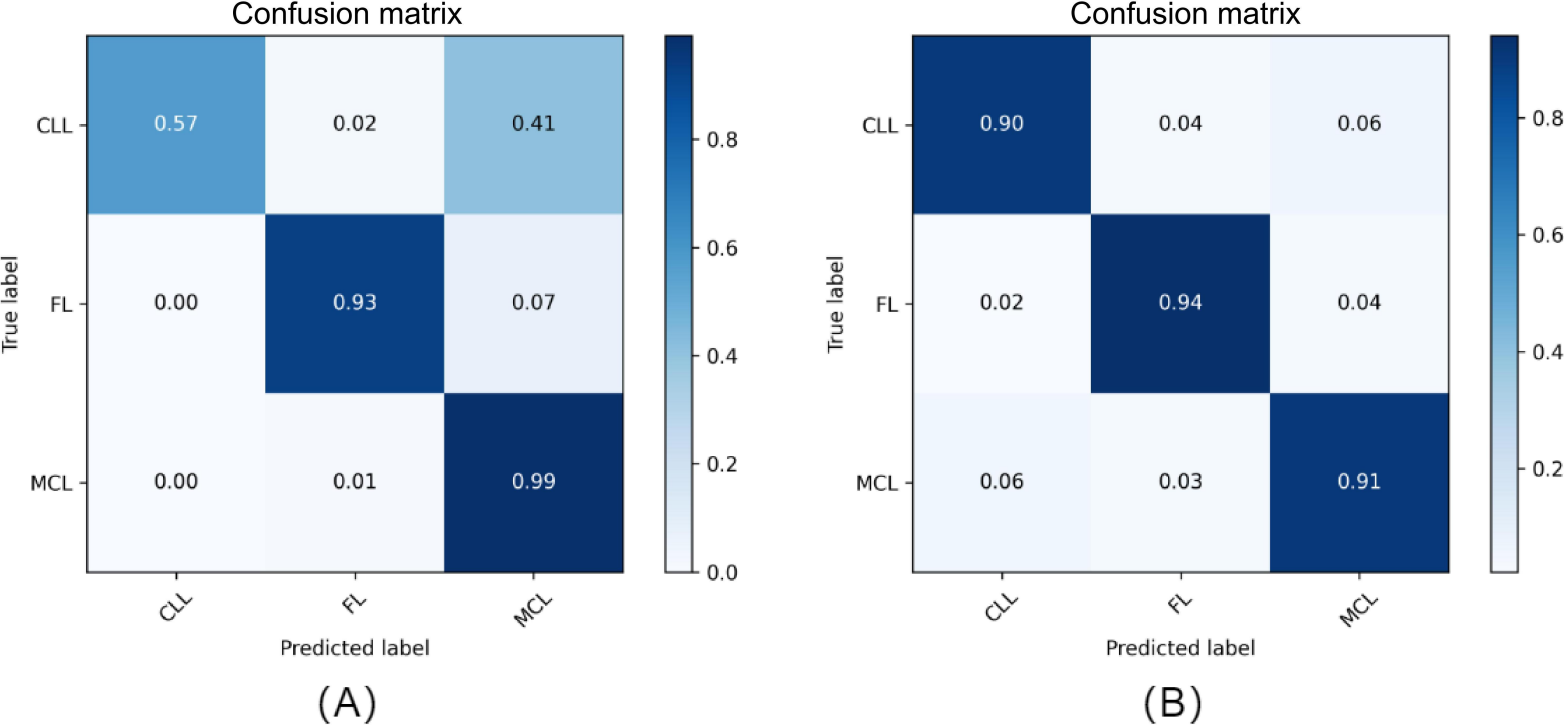
Analysis of attention mechanism and residual network (ResNet) synergy. This figure compares model performance in subtype classification tasks: (A) results of a traditional convolutional neural network and (B) results after incorporating attention mechanisms and ResNet, showing significant improvement in accuracy. CLL: chronic lymphocytic leukemia; FL: follicular lymphoma; MCL: mantle cell lymphoma.

Integrating attention mechanisms with ResNet for feature extraction and classification markedly enhanced model performance. Figure 3 shows the results of lymphoma subtype classification using the combined model. Incorporating the attention mechanism enabled the network to concentrate on diagnostically relevant image regions, thereby reducing errors in differentiating morphologically similar subtypes. Meanwhile, the residual structure of ResNet effectively mitigated the vanishing gradient problem and facilitated the learning of complex image features. Compared with traditional CNNs, the classification accuracy of the proposed deep learning model increased from 81.04% (1013/1250) to 91.04% (1138/1250), representing an improvement of approximately 10 percentage points.

### Evaluation of Model Stability and Generalization Capability

**Figure 4.**
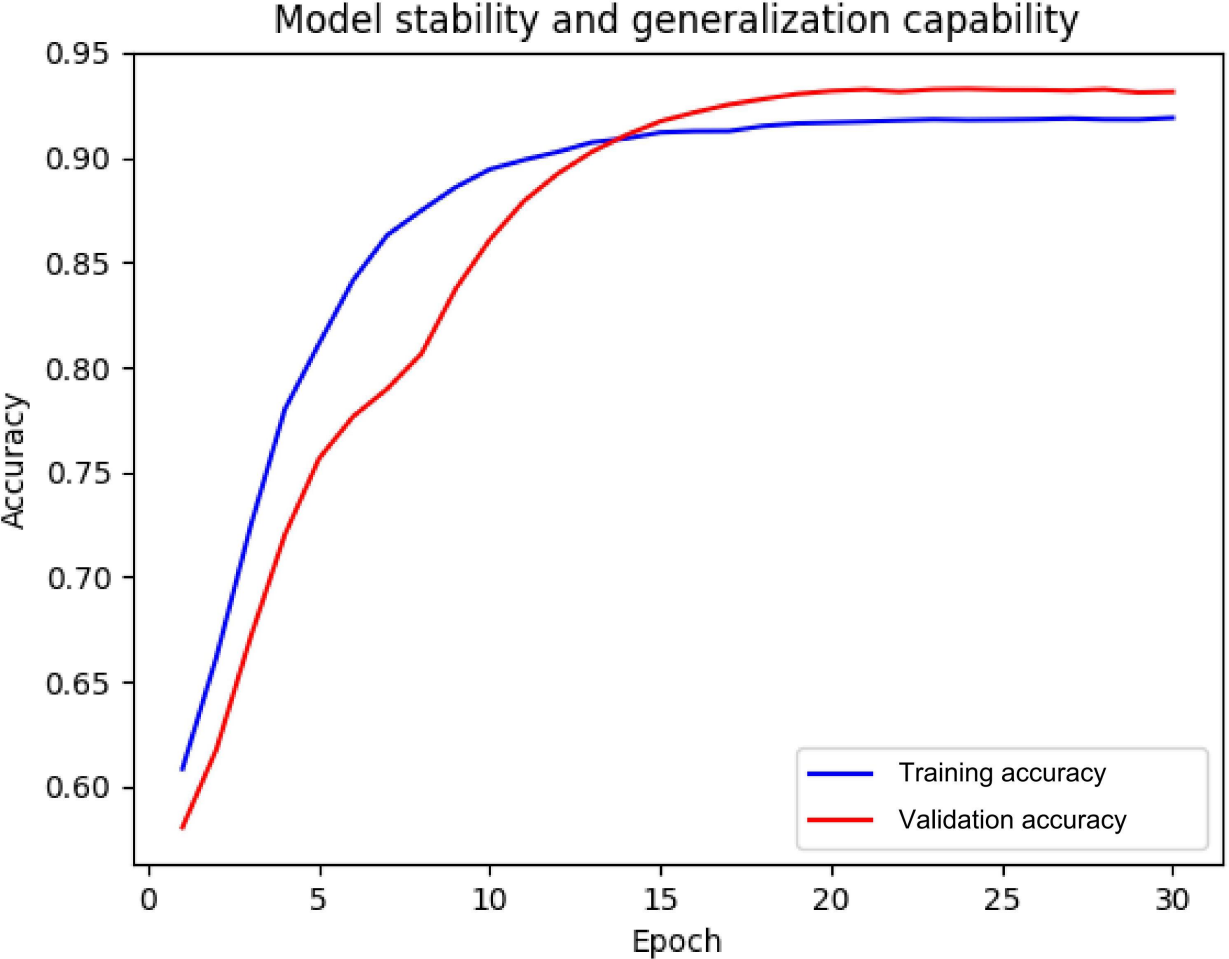
Analysis of model stability and generalization capability. This figure shows the accuracy changes during 5-fold cross-validation. The blue line represents the training set, and the red line represents the validation set. The figure shows stable and minimal fluctuations in model performance across different data splits.

To evaluate the stability and generalization capability of the model, 5-fold cross-validation was performed. The model demonstrated high consistency across the 5 independently partitioned training-validation subsets with only minor fluctuations in accuracy (Figure 4). During cross-validation (N=10,750 samples), the model achieved an average accuracy of 90% (9675/10,750), confirming its robustness and reliability. Even under small-sample conditions, the model maintained high predictive accuracy, indicating good generalization ability and strong resistance to overfitting.

### Analysis of Lymphoma Subtype Classification Results

**Figure 5.**
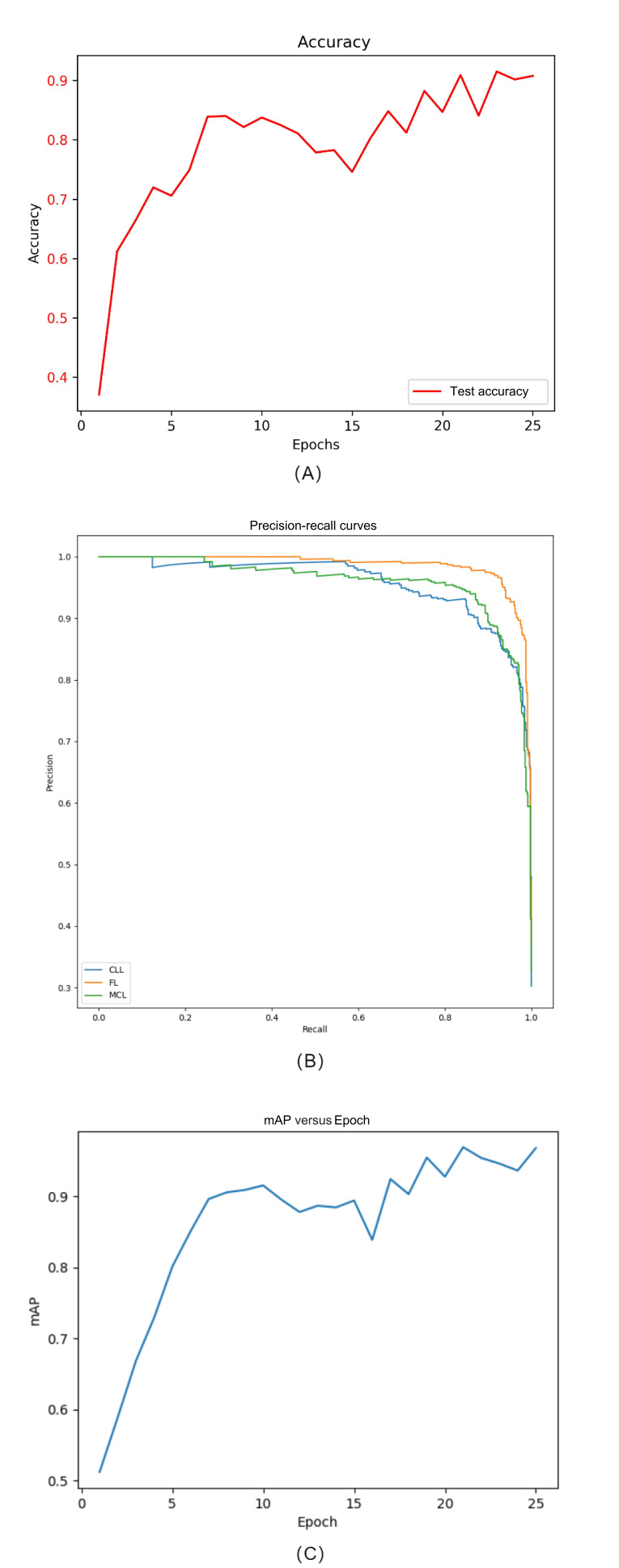
Evaluation of lymphoma subtype classification performance. This figure shows the model’s performance in subtype classification, including (A) accuracy, (B) precision-recall curve, and (C) mean average precision (mAP), reflecting comprehensive performance in multiclass classification tasks. CLL: chronic lymphocytic leukemia; FL: follicular lymphoma; MCL: mantle cell lymphoma.

To evaluate the performance of the deep learning model in lymphoma subtype classification, classification results on the test set were analyzed using accuracy, precision-recall curves, and mAP as evaluation metrics. The results showed that the model achieved a classification accuracy of 98% (1225/1250) when distinguishing FL, chronic lymphocytic leukemia, and MCL. Additionally, the precision-recall curve demonstrated a high AUC, and the mAP reached 97%, indicating strong overall classification performance. Multiclass analysis revealed that most classification errors occurred between subtypes with similar morphological characteristics, such as FL and MCL (Figure 5). The incorporation of a feature enhancement module significantly improved the model’s ability to extract critical features from key regions, leading to a nearly 5% increase in classification performance.

Under 5-fold cross-validation (total sample size: n=10,750), the model achieved an average classification accuracy of 90% (9675/10,750; SD 1.9%), an *F*_1_-score of 90% (9673/10750; SD 2.3%), and an AUC of 0.95 (SD 0.015) across the 3 lymphoma subtypes, all statistically significant (=0.004). In terms of subtype performance, the model demonstrated the highest sensitivity for FL at 94.2% (393/417), followed by chronic lymphocytic leukemia at 90.6% (378/417), whereas the performance for MCL was relatively lower at 87.3% (363/416), which may be attributed to the blurred boundaries and higher heterogeneity typically observed in MCL images. As this study primarily focused on model architecture and performance evaluation, interpretability tools such as gradient-weighted class activation mapping were not incorporated. Future work will include model interpretability analyses to enhance its clinical applicability.

To evaluate the superiority of the proposed model across different deep learning architectures, we systematically compared the fusion model with several mainstream CNN-based frameworks (Table S3 in Multimedia Appendix 1). The results showed that traditional fully convolutional networks and SegNet underperformed in the detection of small lesions and boundary delineation, with overall accuracies of 81.04% (1013/1250) and 83.52% (1044/1250), respectively—significantly lower than those of U-Net and its improved variants (*P*=0.006). DeepLabv3+ achieved improved segmentation accuracy (dice coefficient=83.9%) but suffered from increased training complexity and limited interpretability. In contrast, U-Net demonstrated stable performance in both segmentation and classification tasks (dice coefficient=85.5%; accuracy=1099/1250, 87.92%). The further incorporation of attention mechanisms and ResNet led to continuous performance gains, and the final fusion model achieved the best results in segmentation dice coefficient, classification accuracy, and AUC (dice coefficient=89.7%; accuracy=1150/1250, 92%; AUC=0.95), with statistically significant differences (*P*<). These findings indicate that the proposed fusion model provides a significant advantage in multisubtype classification and grading tasks.

### Comparative Analysis With Traditional Pathological Diagnosis Methods

Compared to traditional pathologists’ manual slide review methods, the deep learning model in this study demonstrated significant advantages in classification and grading tasks of lymphoma pathology images. Figure S7 in Multimedia Appendix 1 shows a comparison of accuracy between the deep learning model and the average accuracy of pathologists. Under conditions involving small sample sizes or diagnostically complex subtypes, the model maintained consistent performance and achieved higher classification accuracy than pathologists. Particularly in images with rich details or blurred edges, the deep learning model reduced human error and improved diagnostic efficiency, highlighting its potential application in clinical practice. To ensure fairness in comparison, all participating pathologists were mid- to senior-level professionals with more than 5 years of diagnostic experience in hematopathology (Figure S7 in Multimedia Appendix 1). Diagnoses were conducted in accordance with the World Health Organization classification guidelines and the expert consensus on pathological diagnosis of lymphoma. Each pathologist independently reviewed the same set of blinded slide images, and no communication was allowed during the review process. Final diagnostic results were determined through majority consensus. This selection protocol was designed to reflect the average diagnostic level of experienced pathologists in routine clinical practice.

### Model Optimization and Accuracy Improvement Strategies

**Figure 6.**
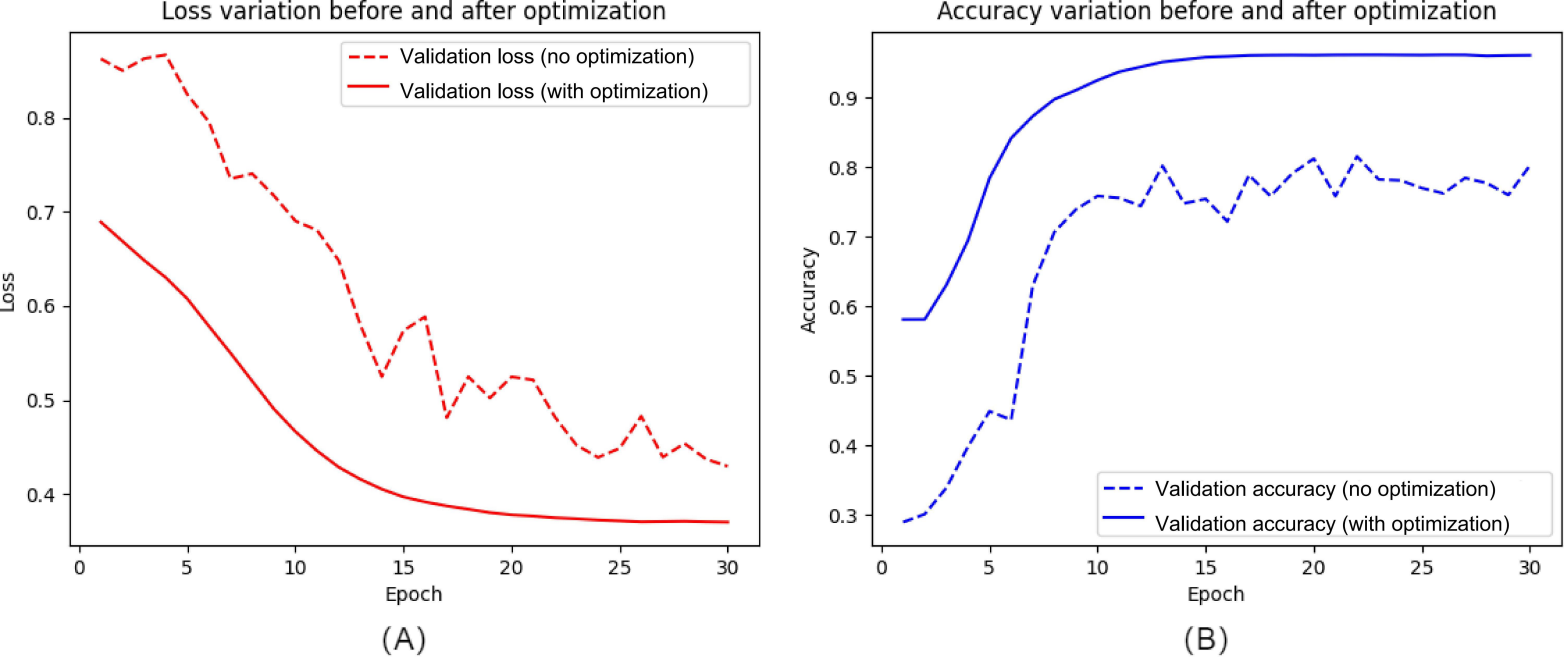
Effects of model optimization on accuracy improvement. This figure illustrates the impact of optimization strategies (learning rate decay, data augmentation, and dropout regularization) on model training: (A) changes in the loss function before and after optimization and (B) changes in accuracy before and after optimization, showing improved accuracy.

To improve model accuracy and stability, a series of optimization strategies were implemented, including learning rate decay, data augmentation, and dropout regularization, which significantly improved model performance. Figure 6 illustrates the performance changes of the model under different optimization strategies. The model gained greater diversity during training through the use of data augmentation, effectively improving its generalization capability. The incorporation of dropout layers effectively mitigated overfitting by reducing model reliance on specific neurons, allowing the network to maintain high accuracy on new datasets.

## Discussion

### Principal Findings

This study explored the application of deep learning techniques, particularly the U-Net model, in classifying and grading lymphoma pathological subtypes. The results showed that deep learning models significantly improved the efficiency and accuracy of automated lymphoma diagnosis. By integrating U-Net for image segmentation with ResNet for feature extraction, a robust diagnostic system was developed capable of differentiating Hodgkin lymphoma from non-Hodgkin lymphoma and accurately predicting lesion grades. Notably, the proposed deep learning model demonstrated significant advantages in segmentation accuracy, classification accuracy, and model stability. These results validated the potential of deep learning in medical image analysis, especially in disease diagnosis and clinical decision support systems.

U-Net exhibited outstanding performance in image segmentation tasks. Its precision in delineating lesion regions highlighted its ability to process complex pathological features. Lymphoma lesion regions often display irregular morphology and poorly defined boundaries, which limit the effectiveness of conventional manual segmentation methods. U-Net, through the hierarchical feature extraction capabilities of CNNs [15], enabled automated and accurate segmentation of lesion areas while minimizing subjective variability. The model achieved an accuracy exceeding 85.04% (1063/1250) on the test set, significantly improving the efficiency of lesion recognition and extraction [9].

However, despite the excellent performance of the deep learning model, several challenges were encountered, particularly regarding data scarcity and annotation quality. The acquisition of pathological images of lymphoma remains difficult, particularly for high-quality annotated data, which may impact model training effectiveness and generalization ability [7]. To address this issue, this study used strategies such as data augmentation and cross-validation [18] to improve the robustness and accuracy of the model. Nevertheless, inconsistencies in data quality, particularly the subjective differences among pathologists during annotation [19], may still affect model training and prediction. Future work could benefit from the development of standardized annotation protocols and the construction of larger, high-quality datasets to further refine model performance.

To optimize model accuracy and stability, several techniques were incorporated, including attention mechanisms, ResNet-based residual learning, and comprehensive data augmentation. The use of attention modules enabled the model to more precisely focus on diagnostically relevant regions within the pathological images, thereby reducing the influence of irrelevant features and enhancing subtype classification accuracy. ResNet’s residual structure effectively mitigated the common issue of vanishing gradients in deep networks, enhancing the model’s ability to learn complex pathological features. These optimization strategies significantly improved the model’s performance in lymphoma subtype classification, achieving a classification accuracy of 92% (1150/1250) and a grading accuracy of 89.04% (1113/1250).

Compared to traditional pathological diagnostic methods, deep learning models offer distinct advantages. Traditional pathology relies on pathologists’ experience and technical expertise, making the diagnostic process vulnerable to subjectivity and relatively inefficient when analyzing complex histological images [20]. In contrast, deep learning models can process large volumes of images quickly and provide efficient and objective diagnostic results [21]. Comparative analysis with manual slide review by pathologists revealed that deep learning models exhibited higher accuracy in handling complex and detail-rich images. Specifically, deep learning models reduced human error and improved diagnostic efficiency in lesion segmentation and subtype differentiation [6,22,23].

Despite the robust performance of deep learning models, several technical limitations remain. First, deep learning models’ “black-box” nature presents a significant challenge, especially in clinical settings, where physicians tend to favor interpretable diagnostic methods. Future research should focus on enhancing model interpretability by using visualization techniques and traceability analysis, allowing clinicians to better understand the rationale behind model predictions. Second, although the model achieved high accuracy in feature extraction and classification tasks, minor errors may still occur in subtype classification and grading, particularly when morphological differences between subtypes are subtle. Improving the model’s performance in analyzing complex pathological images remains a key direction for future research.

In summary, this study confirmed the immense potential of deep learning technologies in lymphoma pathological image analysis and provides robust technical support for the automated diagnosis of clinical lymphoma. With ongoing advancements in computational methods, the application of deep learning in medical imaging analysis will become more extensive and profound. Such progress will play a vital role in developing personalized treatment plans, evaluating therapeutic efficacy, and improving patient survival rates, thereby providing substantial clinical value. The successful application of the proposed approach not only accelerated the diagnostic workflow but also provided clinicians with precise and efficient diagnostic tools, promoting greater automation and enhanced analytical capability in pathological image analysis.

### Conclusions

This study demonstrated the significant potential of deep learning techniques, particularly the U-Net model, in lymphoma pathological subtype classification and grading. By combining U-Net for image segmentation and ResNet for feature extraction, an efficient and accurate diagnostic framework was developed capable of distinguishing Hodgkin lymphoma from non-Hodgkin lymphoma and precisely predicting the grades of lesions. The proposed model achieved strong performance in segmentation accuracy, classification accuracy, and model stability, validating the potential of deep learning technologies in medical imaging analysis, especially in disease diagnosis and clinical decision support.

As the core of this research, the U-Net model exhibited exceptional performance in segmenting pathological regions, effectively addressing challenges such as irregular morphology and blurred boundaries in lymphoma tissues. These capabilities significantly improved segmentation efficiency and accuracy, providing high-quality input data for subsequent classification and grading tasks. Additionally, integrating attention mechanisms and ResNet further optimized feature extraction and classification capabilities, achieving a classification accuracy of 92% (1150/1250) and a grading accuracy of 89.04% (1113/1250).

Despite the encouraging results, certain limitations remain. The limited availability of annotated lymphoma pathological images and inconsistencies in labeling quality may affect the model’s training effectiveness and generalizability. Although data augmentation and cross-validation were used to improve robustness, variability in annotation and the scarcity of high-quality labeled data continue to pose challenges. In addition, the deep learning model’s “black-box” nature limits its interpretability, which affects clinical acceptance and the transparency of decision-making. Moreover, the dataset used in this study was primarily derived from publicly available databases with a relatively limited sample size and without multicenter or real-world clinical validation, which may introduce sampling bias and increase the risk of overfitting. The model also exhibited relatively lower recognition performance for morphologically ambiguous subtypes such as MCL, suggesting that further optimization is needed to address highly heterogeneous lesions. Future work should focus on expanding the dataset with multicenter cohorts, integrating the model into digital pathology workflows as a clinical decision support tool, enhancing interpretability through visualization-based modules, and addressing ethical and regulatory challenges to ensure the safe and responsible application of AI technologies in health care settings.

In summary, this study demonstrated the outstanding performance of U-Net–based deep learning models in analyzing lymphoma pathological images. These techniques significantly improved diagnostic efficiency and accuracy while offering clinicians effective and reliable decision support tools. As AI technology continues to evolve, deep learning models are expected to play an increasingly important role in medical imaging analysis, offering robust support for developing personalized treatment plans and enhancing patient outcomes, thereby advancing medical imaging analysis toward greater automation and intelligence as shown in Multimedia Appendix 2.

## Supplementary material

10.2196/72679 Multimedia Appendix 1 Supplementary figures and tables.

10.2196/72679 Multimedia Appendix 2 Visual abstract.

## Abbreviations

AI: artificial intelligence
AUC: area under the curve
CNN: convolutional neural network
FL: follicular lymphoma
mAP: mean average precision
MCL: mantle cell lymphoma
ResNet: residual network
TCGA: The Cancer Genome Atlas
TCIA: The Cancer Imaging Archive

## References

[1] Pichler AS, Amador C, Fujimoto A (2025). Advances in peripheral T cell lymphomas: pathogenesis, genetic landscapes and emerging therapeutic targets. Histopathology.

[2] Al-Maghrabi H, Al-Maghrabi J (2024). Non-Hodgkin’s primary lymphoma involving the genitourinary tract: histopathological experience from two tertiary hospitals, western region, Saudi Arabia. Am J Clin Exp Urol.

[3] Qiu L, Medeiros LJ, Li S (2025). High-grade B-cell lymphomas: double hit and non-double hit. Hum Pathol.

[4] Doeleman T, Hondelink LM, Vermeer MH, van Dijk MR, Schrader AM (2023). Artificial intelligence in digital pathology of cutaneous lymphomas: a review of the current state and future perspectives. Semin Cancer Biol.

[5] Kuker RA, Lehmkuhl D, Kwon D (2022). A deep learning-aided automated method for calculating metabolic tumor volume in diffuse large B-cell lymphoma. Cancers (Basel).

[6] Haghofer A, Fuchs-Baumgartinger A, Lipnik K (2023). Histological classification of canine and feline lymphoma using a modular approach based on deep learning and advanced image processing. Sci Rep.

[7] Naji H, Sancere L, Simon A (2024). HoLy-Net: segmentation of histological images of diffuse large B-cell lymphoma. Comput Biol Med.

[8] Yang YF, Zhao E, Shi Y, Zhang H, Yang YY (2024). Multicenter investigation of preoperative distinction between primary central nervous system lymphomas and glioblastomas through interpretable artificial intelligence models. Neuroradiology.

[9] Shi T, Jiang H, Wang M, Diao Z, Zhang G, Yao YD (2023). Metabolic anomaly appearance aware U-net for automatic lymphoma segmentation in whole-body PET/CT scans. IEEE J Biomed Health Inform.

[10] Weisman AJ, Kieler MW, Perlman S (2020). Comparison of 11 automated PET segmentation methods in lymphoma. Phys Med Biol.

[11] Isavand P, Aghamiri SS, Amin R (2024). Applications of multimodal artificial intelligence in non-Hodgkin lymphoma B cells. Biomedicines.

[12] Steinbuss G, Kriegsmann M, Zgorzelski C (2021). Deep learning for the classification of non-Hodgkin lymphoma on histopathological images. Cancers (Basel).

[13] Tran KA, Kondrashova O, Bradley A, Williams ED, Pearson JV, Waddell N (2021). Deep learning in cancer diagnosis, prognosis and treatment selection. Genome Med.

[14] Schmidt-Barbo P, Kalweit G, Naouar M (2024). Detection of disease-specific signatures in B cell repertoires of lymphomas using machine learning. PLoS Comput Biol.

[15] Jiang C, Chen K, Teng Y (2022). Deep learning-based tumour segmentation and total metabolic tumour volume prediction in the prognosis of diffuse large B-cell lymphoma patients in 3D FDG-PET images. Eur Radiol.

[16] Jiang H, Diao Z, Shi T (2023). A review of deep learning-based multiple-lesion recognition from medical images: classification, detection and segmentation. Comput Biol Med.

[17] Hamdi M, Senan EM, Jadhav ME, Olayah F, Awaji B, Alalayah KM (2023). Hybrid models based on fusion features of a CNN and handcrafted features for accurate histopathological image analysis for diagnosing malignant lymphomas. Diagnostics (Basel).

[18] Srisuwananukorn A, Salama ME, Pearson AT (2023). Deep learning applications in visual data for benign and malignant hematologic conditions: a systematic review and visual glossary. haematol.

[19] Huang Z, Guo Y, Zhang N (2022). Multi-scale feature similarity-based weakly supervised lymphoma segmentation in PET/CT images. Comput Biol Med.

[20] Schoenpflug LA, Chatzipli A, Sirinukunwattana K (2025). Tumour purity assessment with deep learning in colorectal cancer and impact on molecular analysis. J Pathol.

[21] Huang H, Yan Z, Li B (2024). *LungPath*: artificial intelligence-driven histologic pattern recognition for improved diagnosis of early-stage invasive lung adenocarcinoma. Transl Lung Cancer Res.

[22] Ning X, Liu R, Wang N (2023). Development of a deep learning-based model to diagnose mixed-type gastric cancer accurately. Int J Biochem Cell Biol.

[23] Khalid M, Deivasigamani S, V S, Rajendran S (2024). An efficient colorectal cancer detection network using atrous convolution with coordinate attention transformer and histopathological images. Sci Rep.

