# Supplementary material for: Automated Classification of Lymphoma Subtypes From Histopathological Images Using a U-Net Deep Learning Model: Comparative Evaluation Study
Source: JMIR Med Inform. 2026 Jan 6;14:e72679. doi: 10.2196/72679 (PMC12773696; doi:10.2196/72679)
Supplement: Multimedia Appendix 2 [file medinform-v14-e72679-s002.docx]

**Table S1. Comparative Evaluation of CNN Architectures for Medical Image Segmentation.**

| Architecture | Strengths | Weaknesses | Suitability for Medical Imaging |
| --- | --- | --- | --- |
| FCN | Fast inference; Simple architecture | Low precision on small objects and boundaries | Limited – struggles with fine structure |
| SegNet | Good for semantic segmentation; Moderate computational cost | Information loss during upsampling | Moderate – acceptable on coarse segmentation |
| DeepLabV3+ | Multi-scale context extraction; High accuracy | High computational complexity; Difficult to interpret | Good – strong on large lesions |
| Proposed (U-Net + ResNet + Attention) | Strong edge detection; Stable training; High interpretability | Slightly higher training time; Requires careful parameter tuning | Excellent – designed for fine-grained pathology images |

**Table S2. Performance Comparison Between Raw and Preprocessed Images in Segmentation Tasks.**

| Image Type | Segmentation Accuracy (%) | Edge Continuity | Boundary Sharpness | Training Convergence Speed | Error Rate (%) |
| --- | --- | --- | --- | --- | --- |
| Raw (unprocessed) | 77.1 | Discontinuous, fragmented | Blurred, unclear boundaries | Slow (~150 epochs) | 15.6 |
| Preprocessed | 84.4 | Continuous, well-defined | Sharp, well-visualized edges | Fast (~100 epochs) | 8.2 |

**Table S3. Comparison of the Fusion Model with Several Mainstream CNN-based Frameworks.**

| **Model** | **Segmentation Dice (%)** | **IoU (%)** | **Classification Accuracy (%)** | **Sensitivity (%)** | **Specificity (%)** | **F1 Score** | **AUC** | **Remarks** |
| --- | --- | --- | --- | --- | --- | --- | --- | --- |
| FCN | 78.2 ± 2.5 | 70.5 ± 3.1 | 81.0 ± 2.8 | 79.3 | 82.7 | 0.80 | 0.88 | Limited precision on small lesions and boundaries |
| SegNet | 80.1 ± 2.1 | 73.2 ± 2.7 | 83.5 ± 2.4 | 82.0 | 84.9 | 0.82 | 0.90 | Information loss during upsampling |
| DeepLabV3+ | 83.9 ± 1.9 | 76.8 ± 2.2 | 85.7 ± 2.0 | 84.1 | 87.0 | 0.84 | 0.92 | High accuracy but complex and less interpretable |
| U-Net | 85.5 ± 1.7 | 78.6 ± 2.0 | 87.9 ± 1.8 | 86.4 | 89.3 | 0.86 | 0.93 | High segmentation precision, efficient structure |
| U-Net + Attention | 87.6 ± 1.6 | 80.9 ± 1.9 | 90.5 ± 1.6 | 89.1 | 91.8 | 0.88 | 0.94 | Enhanced recognition of key regions |
| U-Net + ResNet | 88.1 ± 1.5 | 81.4 ± 1.8 | 91.2 ± 1.5 | 89.8 | 92.6 | 0.89 | 0.94 | Improved deep feature extraction |
| U-Net + Attention + ResNet (Final Model) | 89.7 ± 1.3 | 83.2 ± 1.6 | 92.0 ± 1.4 | 91.0 | 89.0 | 0.90 | 0.95 | Best overall performance |

**
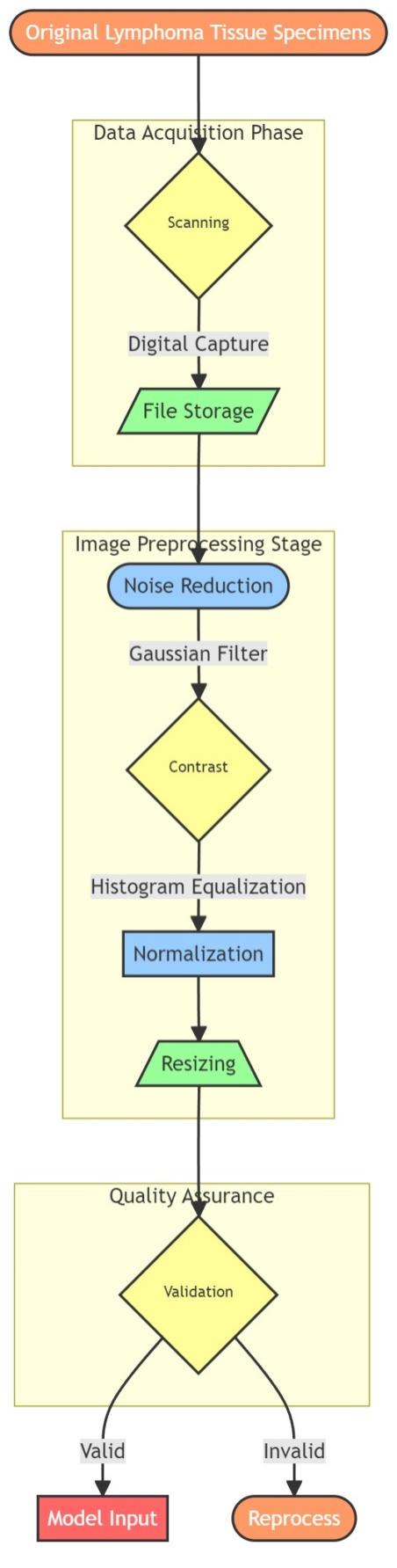
**

**Figure S1. Data Collection and Preprocessing Flowchart.**

Note: This figure illustrates the complete workflow of acquiring and preprocessing lymphoma image data, including noise removal, contrast enhancement, and normalization.

**
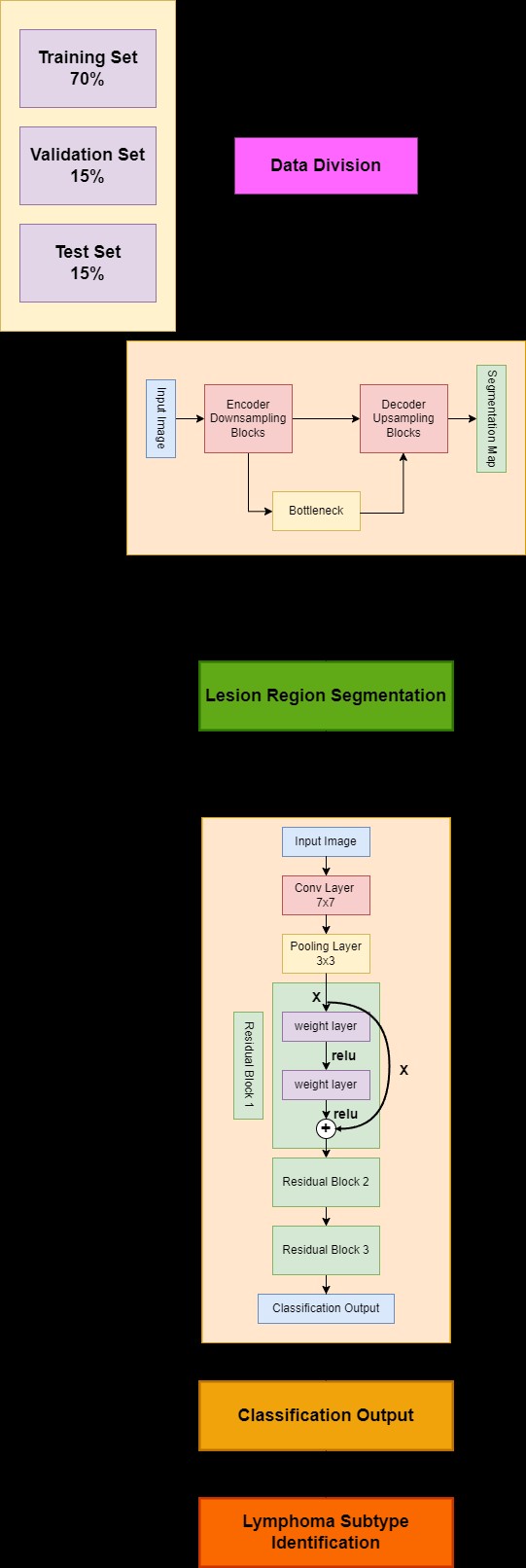
**

**Figure S2. Image Segmentation and Feature Extraction Flowchart.**

Note: This figure demonstrates the integration of U-Net and ResNet models for image segmentation and feature extraction, highlighting how U-Net segments lesion areas and ResNet extracts deep features.

**
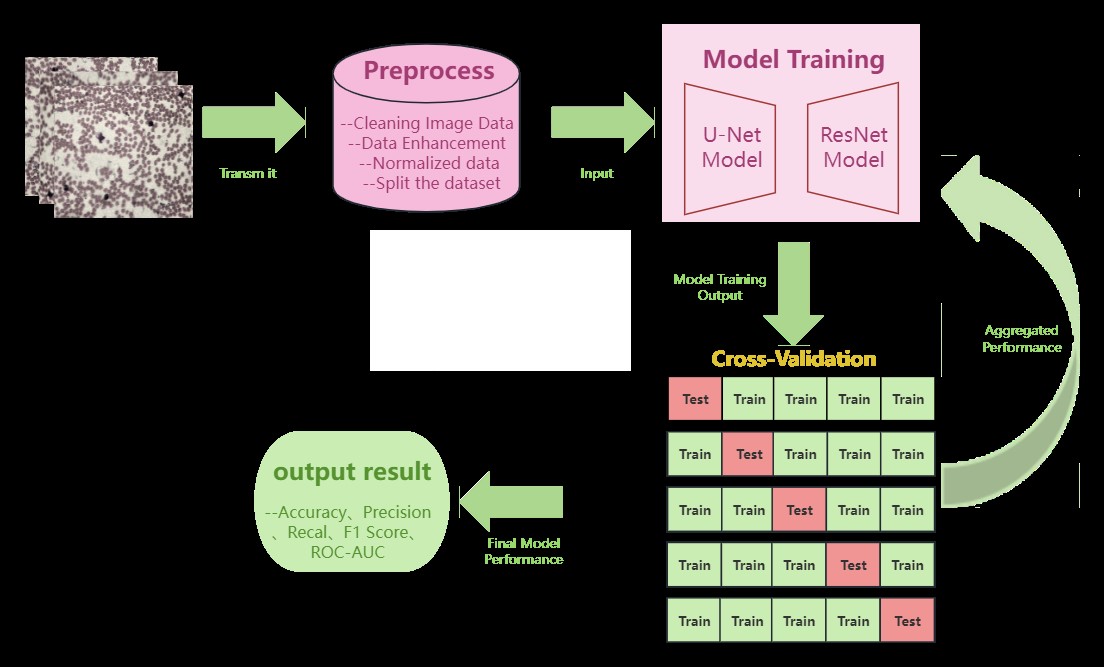
**

**Figure S3. Model Training and Optimization Flowchart.**

Note: This figure shows the entire process of training U-Net and ResNet models, including dataset division, cross-validation, and learning rate decay.

**
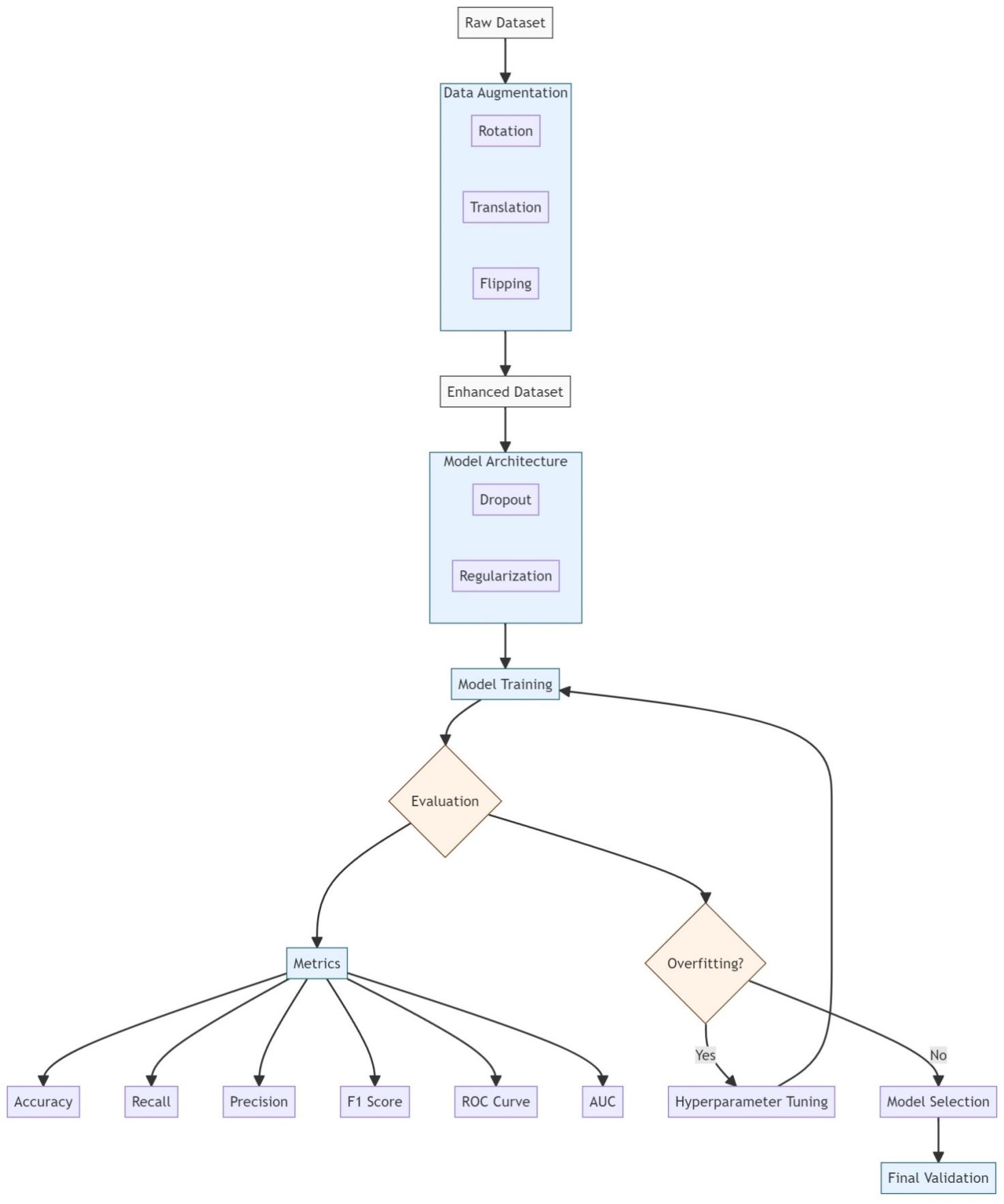
**

**Figure S4. Model Evaluation and Performance Optimization Flowchart.**

Note: This figure outlines the performance evaluation and optimization steps during model training, including dropout layers and data augmentation to improve model stability and robustness.

**
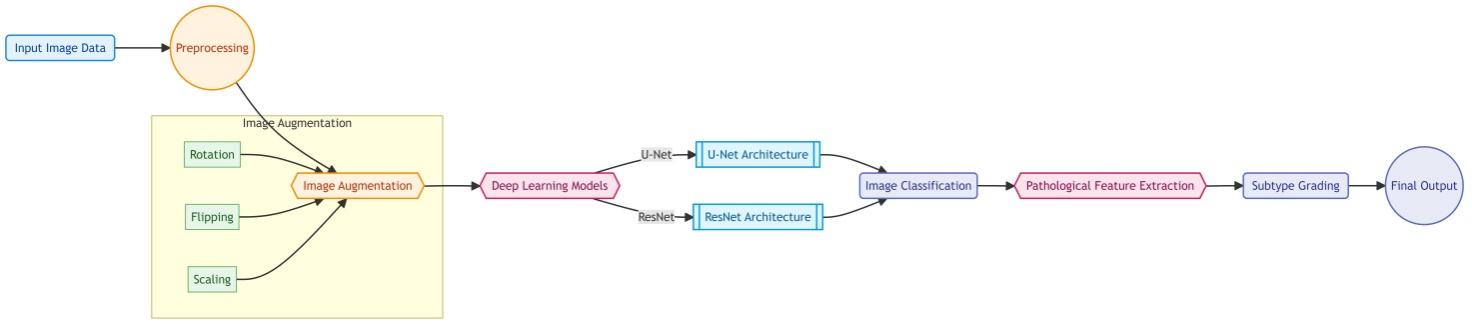
**

**Figure S5. Image Classification and Subtype Grading Analysis Flowchart.**

Note: This figure illustrates the workflow for image classification and subtype grading analysis using a deep learning model based on U-Net and ResNet, emphasizing the integration of pathological features for accurate classification.

**
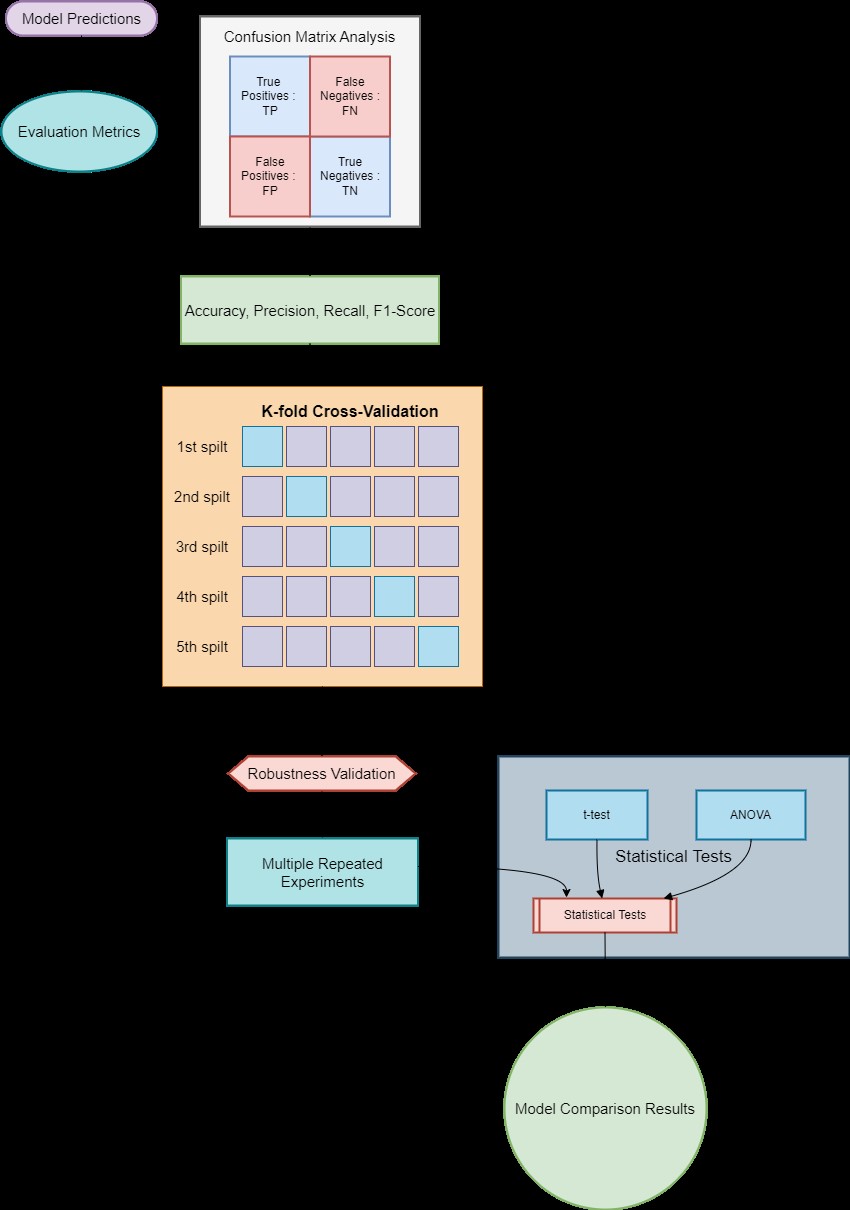
**

**Figure S6. Model Evaluation and Statistical Analysis Flowchart.**

Note: This figure presents model performance evaluation through statistical analysis and cross-validation, employing methods such as confusion matrices and t-tests to analyze differences between models.
